# Automatic and Real-Time Computation of the 30-Seconds Chair-Stand Test without Professional Supervision for Community-Dwelling Older Adults

**DOI:** 10.3390/s20205813

**Published:** 2020-10-14

**Authors:** Antonio Cobo, Elena Villalba-Mora, Rodrigo Pérez-Rodríguez, Xavier Ferre, Walter Escalante, Cristian Moral, Leocadio Rodriguez-Mañas

**Affiliations:** 1 Centre for Biomedical Technology (CTB), Universidad Politécnica de Madrid (UPM), Pozuelo de Alarcón, 28223 Madrid, Spain; antonio.cobo@ctb.upm.es (A.C.); xavier.ferre@ctb.upm.es (X.F.); walter.escalante@ctb.upm.es (W.E.); cristian.moral@ctb.upm.es (C.M.); 2 Centro de Investigación Biomédica en Red en Bioingeniería, Biomateriales y Nanomedicina (CIBER-BBN), 28029 Madrid, Spain; 3 Fundación para la Investigación Biomédica, Hospital de Getafe, Getafe, 28905 Madrid, Spain; rprodrigo@salud.madrid.org; 4 Servicio de Geriatría, Hospital de Getafe, Getafe, 28905 Madrid, Spain; leocadio.rodriguez@salud.madrid.org; 5 Centro de Investigación Biomédica en Red en Fragilidad y Envejecimiento Saludable (CIBER-FES), 28029 Madrid, Spain

**Keywords:** frailty syndrome, sit-to-stand, 30-s chair stand test, wearable sensors, signal processing

## Abstract

The present paper describes a system for older people to self-administer the 30-s chair stand test (CST) at home without supervision. The system comprises a low-cost sensor to count sit-to-stand (SiSt) transitions, and an Android application to guide older people through the procedure. Two observational studies were conducted to test (i) the sensor in a supervised environment (*n* = 7; m = 83.29 years old, sd = 4.19; 5 female), and (ii) the complete system in an unsupervised one (*n* = 7; age 64–74 years old; 3 female). The participants in the supervised test were asked to perform a 30-s CST with the sensor, while a member of the research team manually counted valid transitions. Automatic and manual counts were perfectly correlated (Pearson’s r = 1, *p* = 0.00). Even though the sample was small, none of the signals around the critical score were affected by harmful noise; p (harmless noise) = 1, 95% CI = (0.98, 1). The participants in the unsupervised test used the system in their homes for a month. None of them dropped out, and they reported it to be easy to use, comfortable, and easy to understand. Thus, the system is suitable to be used by older adults in their homes without professional supervision.

## 1. Introduction

The present paper describes a system for older people to self-administer the 30-s chair stand test (CST) at home without supervision. The system comprises a low-cost sensor that automatically detects and counts sit-to-stand (SiSt) transitions in real-time, and a home care application that guides older people through the whole procedure. Since using novel technologies is not a trivial issue for older adults, we studied whether such a system would be able to match older people’s abilities and expectations, so they can use it without supervision. The 30-s CST is a medical exam used to assess older adults’ lower-limb strength [1]. It requires a subject to spend thirty seconds repeatedly standing up from, and sitting down on, a chair, with his arms folded on his chest, as fast as possible [1]. The number of times the said subject reached an upright position is then taken as a proxy to lower-limb strength [1]. It is used in combination with other medical exams to assess the functional status of older people. Depending on the results of such a functional assessment, older people may be diagnosed as robust, pre-frail, or frail [2]. Frailty is a state of increased vulnerability to low power stressors, leading to difficulties in maintaining homeostasis that increases the risk of disability and other adverse outcomes, such as falls, hospitalization, permanent institutionalization, and death [3,4,5,6]. In fact, frailty is a major predictor of disability, with frail elders showing twice the risk of disability than non-frail older adults [7].

Disability is one of the major challenges for elderly care, because improvements in life expectancy are not coming together with similar improvements in impairment-free life expectancy (IFLE); on the contrary, a decline in the latter has even been observed in some countries [8]. Disability imposes a heavy psychological and economic burden on older people and their relatives over a long time; fortunately, frailty, which may precede the development of disability by several years [2], can be reversed [9,10,11]. Hence the paramount role of frailty detection in the prevention of disability. Several models have been proposed to explain frailty, with two of them prevailing as major approaches, namely, Rockwood’s deficit accumulation model [12,13,14] and Fried’s phenotypic model [2]. The latter is the most widespread, and identifies the following markers of frailty: (i) weight loss, (ii) exhaustion, (iii) weakness, (iv) slowness, and (v) low physical activity [2]. An older adult is classified as pre-frail if he tests positive to one or two of the frailty components in the phenotypic model, and as frail if he tests positive to three or more of them; otherwise he is classified as robust [2] (see Table 1).

Frail older adults can reduce their levels of frailty, and even be restored back to robustness, with exercise-based interventions, especially if combined with early diagnosis and continuity of care [9,11,15]. Many different instruments are currently involved in the diagnosis and assessment of frailty. For instance, hand grip strength is used to assess weakness, in fact, the data in the original study of Fried et al. came from hand grip strength measurements [2], but it has also been explored as a measurement of overall frailty on its own; just as the stand up and go (TUG) test, which is a standard test for gait speed [6]. On the other hand, other sources of weakness measurements, such as those based on lower-limb strength, have been observed to be associated with either hand grip strength, gait speed, and even overall frailty [16]. In fact, instruments to assess lower-limb strength, such as the 30-s CST [1], and the STS5 (measuring how long it takes for an older person to repeatedly stand up from a chair five times) [17], are usually included as part of a comprehensive geriatric assessment (CGA). Both early diagnosis and continuity of care require frailty to be frequently assessed in search of early signs of functional decline. For instance, if two consecutive measurements of lower-limb strength taken two weeks apart with the 30-s CST show a decrease bigger that a given threshold, an alarm should be raised. However, it is not feasible to assess every older adult at risk of developing frailty every two weeks with the currently available means to conduct the functional assessment exams. Most of them require the involvement of a specifically trained professional in a geriatrics department to supervise their execution, and compute their corresponding scores. Obviously, a geriatrics department in specialized care cannot afford to undertake such a screening task. In fact, they should be focusing on taking care of the most severe cases. As a result, older people do not have their functional capacity assessed for early signs of frailty very often.

Automatic sensors that do not require the involvement of any specifically trained personnel could help to alleviate this problem. On the one hand, a single staff member in a geriatrics department could supervise several tests on multiple patients simultaneously. On the other hand, and probably involving a bigger potential impact, general practitioners or nurses in primary care could add functional assessment to routine follow-ups of their older adult patients. The potential benefits of such automatic sensors become even more remarkable within the current context of the COVID-19 pandemic. Older people are at greater risk of developing severe complications and dying from COVID-19 [18]. Therefore, they have been advised to carefully comply with distancing measures to lower the risk of transmission. However, distancing measures favor social isolation and sedentary behaviors. Such sedentary behaviors increase the risk of developing frailty [19], and have been hypothesized to persist and become habits, based on observations from previous natural disasters, in particular, from the three years following the 2011 earthquake and tsunami in East Japan [20]. In such a scenario (i.e., fewer visits to the doctor and increased risk of frailty due to sedentary behavior) older adults would benefit from having automatic sensors to conduct functional assessment exams at home, on their own or helped by their care givers. In the particular case of the 30-s CST, the setup is rather simple; it just requires a regular rigid chair to repeatedly stand-up and sit-down, and a timer to control the duration of the test [1]. Nevertheless, a trained professional is required to judge which SiSt transitions are valid and must be added to the final score. Valid SiSt transitions occur when the subject reaches an upright position [1]. However, some older adults suffer from mobility constraints, and upright positions might differ from one subject to another.

A 30-s CST is not a transparent procedure embedded into people’s daily lives. It requires older adults to interrupt their daily activities and go through a specific sequence of actions. Our approach involves a system that comprises an automatic sensor and a home care application to guide older adults through the procedure. One of the determinant factors of such a system is the specifics of the sensing device. According to Millor et al., the study of SiSt and stand-to-sit (StSi) transitions with inertial sensors can be traced back to the mid-1990s [21]; in particular to Kerr et al., in 1994 [22]. Over half of the works that Millor et al. reviewed were based on the assessment of daily life activities [21]. Only a few of them involved the assessment of repeated SiSt/StSi cycles in traditional tests for frailty assessment [21]. While some works relied on the use of multiple devices on different parts of the body, a low number of devices is recommendable to simplify the setup, lower the cost, and eventually improve acceptance and adoption. A very popular approach is to place a single inertial measurement unit (IMU) on the subject’s lower back (L3–L5 region), close to the body center of mass, and take advantage of the quasi-periodic nature of the body movement [23,24]. This approach is exemplified in the works of van Lummel et al. (where the authors apply it to the STS5) [23] and Millor et al. (where the authors apply it to the 30-s CST) [24].

In particular, van Lummel et al. developed a fully automated method of processing repeated sit-to-stand-to-sit (STS) cycles [23]. They used triaxial acceleration and triaxial angular velocity signals from a single IMU device (dynaport) on the lower back to compute trunk pitch-angle and vertical velocity signals. They used the morphological properties of the trunk pitch-angle signal to identify and delimit the sub-phases and transitions in repeated STS cycles. They used the vertical velocity signal to spot and discard failed attempts. However, they do not explain which features of the vertical velocity and which criteria were used to classify an attempt as a failure.

On the other hand, Millor et al. developed a fully automated method to process repeated STS cycles in a 30-s CST [24]. They computed vertical velocity and vertical position from the vertical acceleration signal, from a single IMU device (MTx Orientation Tracker—Xsens Technologies B.V. Enschede, Netherlands) on the lower back. They applied double integration, combined with fourth-level polynomial curve adjustment and cubic splines interpolation. They used the morphological properties of the vertical position signal to identify and delimit complete STS cycles. A complete STS cycle can be found between two minima in the vertical position signal. They also used the MTx’s onboard Kalman filter estimation for the X-orientation, and combined it with the vertical acceleration, the vertical velocity, and the vertical position signals to identify the sub-phases (i.e., impulse, stand up, and sit down) within each STS cycle. In a subsequent paper Millor, Lecumberri, Gómez, Martínez-Ramírez, and Izquierdo argued that they automatically detected failed attempts “... based on a threshold applied to both the time elapsed between a maximum and a minimum of the Z-position and to their difference.” [25], (p. 4). However, they did not clarify what the values for these thresholds were nor how they were computed.

In both cases, the authors complemented their studies by computing multiple kinematic parameters, such as transition duration (TD), maximum and minimum values of the vertical acceleration (max., min., V-acc.), Area Under the Curve (AUC) of V-acc., and roll range, and processed them to obtain additional information beyond the test score [25]. van Lummel et al. were able to identify seat-off and seat-on instants [26], establish a relationship between the subjects’ stand up strategies and their overall muscle strength [27], and compare the sensitivity of stair ascending (SA) and Leg-Extension Power (LEP) to detect age-related changes [28]. When they analyzed the associations between clinical outcomes (both health and functional outcomes) and the functional tests results, they observed stronger associations for their instrumented STS5 test than for manual records [29]. On the other hand, Millor et al. were able to detect differences in frailty status (robust, pre-frail, frail) across different subjects directly from their kinematic parameters [25]. They even identified the set of most informative parameters (i.e., anterior-posterior (AP) orientation range during the Imp phase, maximum vertical acceleration and vertical power peaks during SiSt phase, and total impulse during the StSi phase) [30]. In fact, they claimed that these parameters outperformed the number of completed cycles in the 30-s CST, as a criterion for frailty classification [30].

Other locations for the sensing devices, such as the chest, have also been explored [31]. Recently, in that line, Jovanov, Wrigth, and Ganegoda presented some preliminary results from their automated 30-s CST [32]. Instead of attaching a sensor directly to the subject’s chest, they took advantage of the fact that the 30-s CST requires the subject to fold his arms over his chest, and used the inertial sensors onboard a smartwatch. They used 3D acceleration signals from two different models (Fossil Gen 4 and Polar M600). They obtained excellent reliability between automated and manual counts, with little processing load. However, their experimental subjects were not older adults (12 subjects, mean age: 39.1 y.o.). They did not provide any explanation of the STS cycle identification and delimitation criteria and algorithms, and they did not mention any mechanism to spot and dismiss failed attempts.

Lately, we have explored a different approach ourselves by using an ambient sensor instead of a body-worn sensor [33]. In our study we explored “… the feasibility of using the quasi-periodic nature of the distance between a subject’s back and the chair backrest during a 30-s CST to carry out unsupervised measurements based on readings from a low-cost ultrasound sensor” [33], (p. 3). Our sensor comprised an ultrasound sensing module, an Arduino controller board, and a wireless communications module. All three of them were integrated into our own design for a portable device that the end-users could attach to the backrest of any regular rigid chair. We observed older people to generate very noisy signals. We applied a moving minimum filter to cancel the effects of said noise and an adaptable threshold to tell the difference between sitting and standing regions in the signal. Even though intra-class correlation coefficients showed good levels of reliability between the sensor outcomes and the trained professional’s manual counts, the differences between these outcomes resulted in the performance of some subjects not being correctly classified as average, better than average, or worse than average.

In the present paper we come back to the body-worn sensor approach. We propose to measure the thigh angle with respect to a horizontal plane perpendicular to the direction of gravity (i.e., tilt) with a single device on the subject’s thigh itself, and to use the variation of the angle as the subject stands up and down over time to identify SiSt and StSi transitions. Measurements of the thigh angle from a single device have already been used in previous literature to study SiSt and StSi transitions, mostly to identify different postures and activities (sitting, standing, walking, ramp or stair ascending, etc.) while performing activities of daily living (either in controlled lab settings or in free-living conditions) [34,35,36,37]. However, we have not found any descriptions of an instrumented version of the 30-s CST based on this approach. The accurate estimation of tilt based on IMU readings relies on the fusion of accelerometer and gyroscope data [38]. Smartphones come equipped with IMUs and relatively high computing power. However, smartphone adoption among the geriatric population (i.e., people over 70 years old) is low, especially among low-income and low-education elders, because they use much simpler and cheaper mobile phones. Smartphones would be too expensive, and oversize, for the single purpose of being used as a sensor; since a home kit for frailty monitoring usually comprises multiple sensors, devices of a much lower cost are required. Kalman and complementary filters are the most widespread data fusion methods for IMU-based applications [38]. Kalman filters are computationally expensive and, as stated by Abhayasinghe, Murray, and Sharif Bidabadi, 32-bit microcontrollers with a digital signal processor (DSP) are necessary to run them in real time [39]. Conversely, the algorithm for the complementary filter is much simpler and, even though it involves the computation of an arc tangent, can be run on much cheaper 8-bit microcontrollers in real time [39]. According to Tognetti et al., making use of a simple accelerometer instead of a complete IMU may contribute to further decreasing the complexity and cost of the sensing device [40]; however, the complementary filter still relies on fusing accelerometer and gyroscope data. Fortunately, tilt can be estimated solely from accelerometry if the main contributor to the accelerometer readings is gravity. During a 30-s CST, however, an accelerometer will be exposed to sudden acceleration and deceleration forces when reaching the upright and sitting positions. Thus, the question remains whether the resulting noise will harm the correct identification of valid transitions.

On the other hand, using novel technologies is not a trivial issue for older adults. Moreover, the sensors described above have been tested in controlled settings, under the supervision of their corresponding research teams. We have found no works reporting older adult’s performance when using this kind of automatic sensors on their own. Our approach involves a system that comprises an automatic sensor and a home care application to guide older adults through the procedure. Thus, the question remains whether such a system will match older people’s abilities and expectations so they can use it without supervision. We implemented our own design for a low-cost sensor for counting SiSt transitions, which estimates the thigh angle solely form the readings of a single accelerometer. In addition, we implemented a home care app for Android that guides the older adults throughout the whole procedure. We first tested the sensor in a supervised environment, and then we tested the complete system in an unsupervised one. To test the sensor, we studied the impact of noise by analyzing the statistical significance of the estimated probability of finding harmless noise in a valid SiSt transition. We observed the noise in all the valid transitions in the critical scenario (i.e., test scores around the value used to spot patients not fit enough to remain independent) to be harmless. We then delivered the complete system to seven older adults’ homes for a month, and conducted an acceptability study. The participants reported finding it easy to use, feeling comfortable using it, understanding the features and functionalities of the app, and feeling able to use it on their own.

## 2. Materials and Methods

The sensor was tested in an observational study, described in Section 2.1, where the participants used it while taking a 30-s CST, under the supervision of a trained member of the research team. The home care system was tested in another observational study, described in Section 2.2, where the participants used it to take several 30-s CSTs over the course of a month, at their own homes and without any kind of supervision.

### 2.1. Supervised Validation of the Sensor

#### 2.1.1. Participants

Seven older subjects (age: m = 83.29 years old, sd = 4.19; gender: 5 female and 2 male) were recruited from a pool of participants that expressed a general interest in participating in research studies from the University Hospital of Getafe.

All subjects gave their informed consent for inclusion before they participated in the study. The study was conducted in accordance with the Declaration of Helsinki, and the protocol was approved by the Ethics Committee of the Universidad Politécnica de Madrid on 9 May 2019 (POSITIVE: Maintaining and improving the intrinsic capacity involving primary care and caregivers).

The following inclusion and exclusion criteria were applied:

- A subject COULD ENTER the study if ALL the following INCLUSION CRITERIA applied:
  ◦ The subject is willing and able to give written informed consent for participation in the study.
  ◦ The subject is 70 years old or older.
  ◦ The subject is able to perform the 30-s CST in a safe way.
  ◦ The subject has not been diagnosed with cognitive impairment.
- A subject COULD NOT ENTER the study if ANY of the following EXCLUSION CRITERIA applied:
  ◦ Subjects suffering from any major disability.
  ◦ Subjects suffering from cognitive impairment.

#### 2.1.2. Apparatus

**Figure 1:**
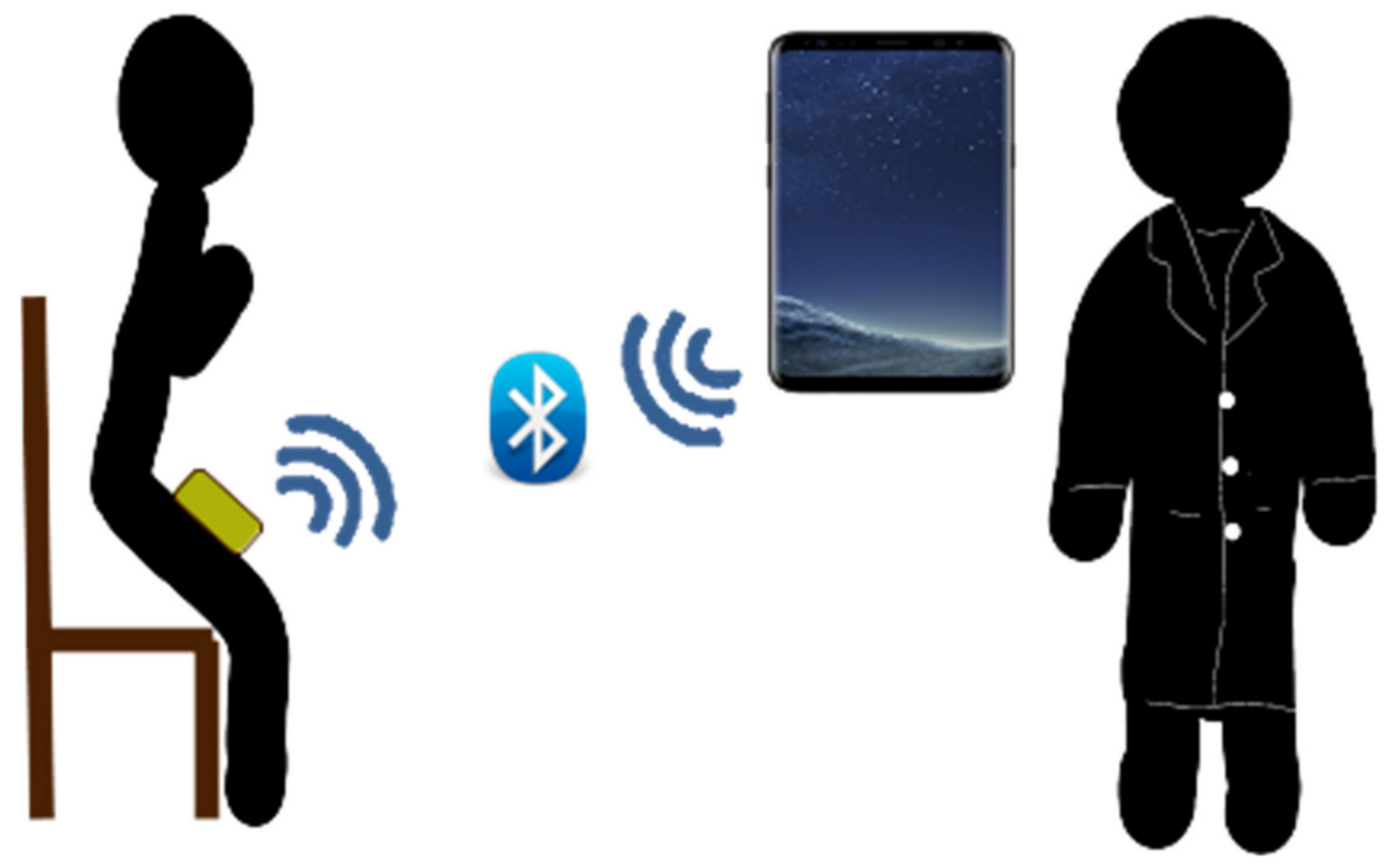
Depiction of the overall experimental setup: The subject stands up from and sits down on a regular rigid chair with the sensing device on one of his thighs; the sensing device is paired with a tablet via Bluetooth; a member of the research team uses the app on board the tablet device to switch the sensor into either calibration or measurement modes, and to visualize the sensor automatic count once the 30-s chair stand test (CST) is over.

**Figure 2:**
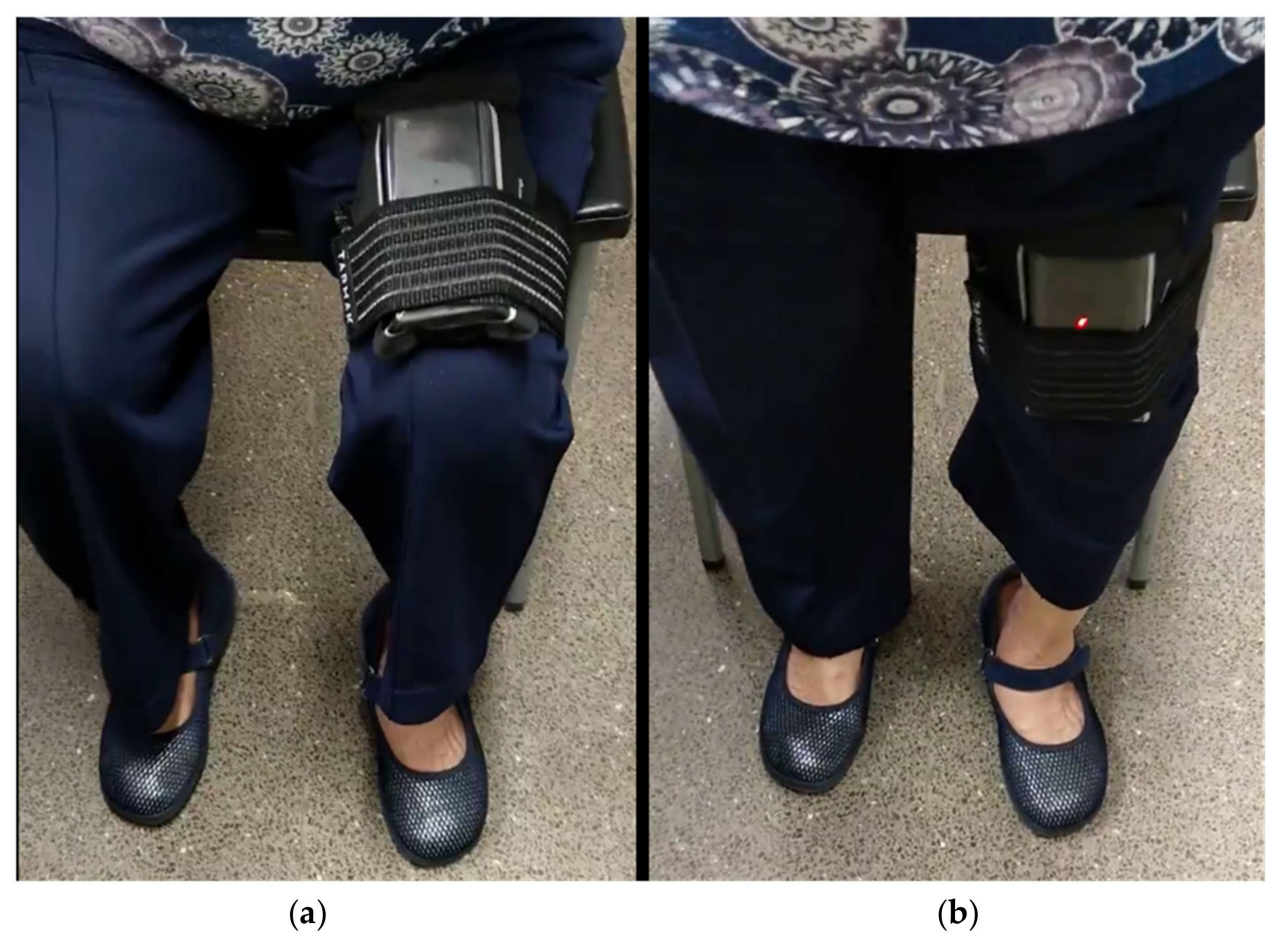
Position and alignment of the sensing device on a subject’s thigh. (**a**) The wearable sensor is placed on the subject’s thigh longitudinally aligned with her femur. The sensor is tightly attached to the subject’s thigh with a Velcro strap to prevent it from sliding. The LED in the sensor is turned off when the subject is sitting; (**b**) and it is turned on every time a valid SiSt transition is detected.

The overall setup is depicted in Figure 1, and consisted of a regular rigid chair with a backrest, an instance of the wearable device under study, and a tablet device. The chair played the same role as usual in any regular 30-s CST. The subjects wore the sensing device on one of their thighs. The sensor is longitudinally aligned with the subject’s femur and tightly attached to her thigh with a Velcro strap as shown in Figure 2. Since the sensor is sensitive to orientation, a green sticker was attached to one of its ends to signal which one has to remain closer to the knee. However, it is not visible in Figure 2, because once the sensor is put in place and secured, the Velcro strap covers it. The tablet hosts an app to control the sensor, and it is paired to the latter via Bluetooth. A member of the research team used the app to switch the sensor into either calibration or measurement mode, and to visualize the sensor automatic count at the end of each 30-s CST. In calibration mode, the readings from the accelerometer in the device are used to compute the thigh angle in both a sitting and an upright static posture, as a measurement of the subject’s mobility constraints; then, the parameters in the automatic count algorithm are set accordingly to a personalized value. In measurement mode, the subject takes the 30-s CST itself, and the accelerometer readings are processed by the automatic count algorithm (aka STS analysis algorithm). Finally, the sensor sends the automatic count to the tablet via Bluetooth once the test is over. Further details about the sensor hardware, the automatic count algorithm, and the tablet app can be found in Section 2.1.2.1, Section 2.1.2.2 and Section 2.1.2.3, respectively.

##### 2.1.2.1. The Wearable Sensor Device

**Scheme 1:**
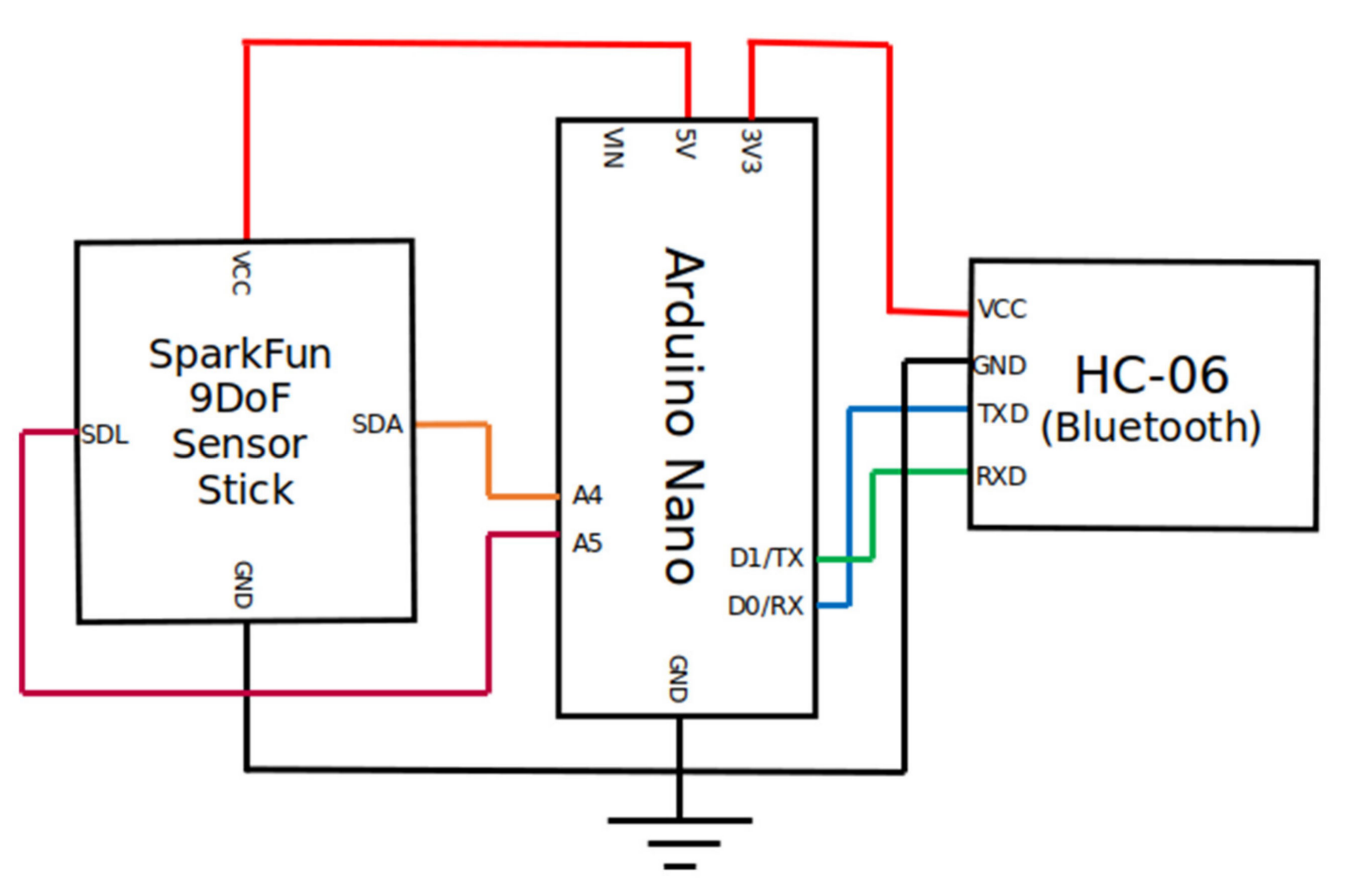
Schematic block diagram of the interconnection between the device components. The Arduino Nano board (center) acts as the control and processing unit, collecting readings from the accelerometer (SparkFun block on the left), computing the estimations of the thigh angle over time, and analyzing the resulting signal. The Arduino Nano board also makes use of the Bluetooth module (HC-06 block on the right) to exchange messages with the external mobile device over a wireless communication channel. The whole device was powered by a 9V 6LP3146 battery. While the Arduino Nano board was directly powered by the battery, the accelerometer and the Bluetooth boards were indirectly powered by connecting them to the Arduino’s 5V and 3.3V DC outputs, respectively. The battery was omitted in this scheme for the sake of clarity.

The device consists of three main building blocks, as shown in Scheme 1. These blocks are, from left to right:

- An accelerometer (a SparkFun 9DoF Sensor Stick board with an LSM9DS1 IMU chip). This SparkFun board comes with a nine degrees of freedom IMU (i.e., it comprises a triaxial accelerometer, a triaxial gyroscope, and a triaxial magnetometer). However, we did not use the gyroscope and the magnetometer because, as explained in Section 2.1.2.2, acceleration readings are enough to compute an estimation of the thigh angle.
- A control and processing unit (an Arduino Nano board with an ATmega328P microcontroller). The Arduino board acts as the processing unit in the device thanks to its onboard micro-controller. Our processing algorithm runs on board the Arduino, and is responsible for collecting the accelerometer readings, computing the estimations of the thigh angle over time, and analyzing the resulting signal to automatically detect and count SiSt and StSi transitions in real time, without storing or transmitting the individual samples.
- A communications unit: (HC-06 Bluetooth 2.0 + EDR module). End users (in this case, the researcher conducting the experiment) control the behavior of the sensing device by interacting with a mobile app in an external tablet device. This communication unit enables wireless communication between the two devices via Bluetooth. The researcher can issue calibration and measurements commands to the sensing device, and the latter automatically sends the results to the tablet once a 30 s-CST is over.

The device is powered by a 9 V battery (6LP3146). However, only the Arduino Nano board was directly powered by this battery. The accelerometer board was powered by connecting it to the Arduino’s 5 V DC output, and the Bluetooth board was indirectly powered via the Arduino as well, by connecting the Bluetooth board to the Arduino’s 3.3V DC output. The device also has an on/off switch, a LED, and a vibrator. The color of the LED helps to tell the difference between calibration mode and measurement mode. Once the device enters into measurement mode, the vibrator tells the subject when to start and stop the test. All these additional elements (the battery and its corresponding case, the on/off switch, the LED, and the vibrator) were omitted in Scheme 1 for the sake of clarity.

##### 2.1.2.2. The STS Analysis Algorithm

**Figure 3:**
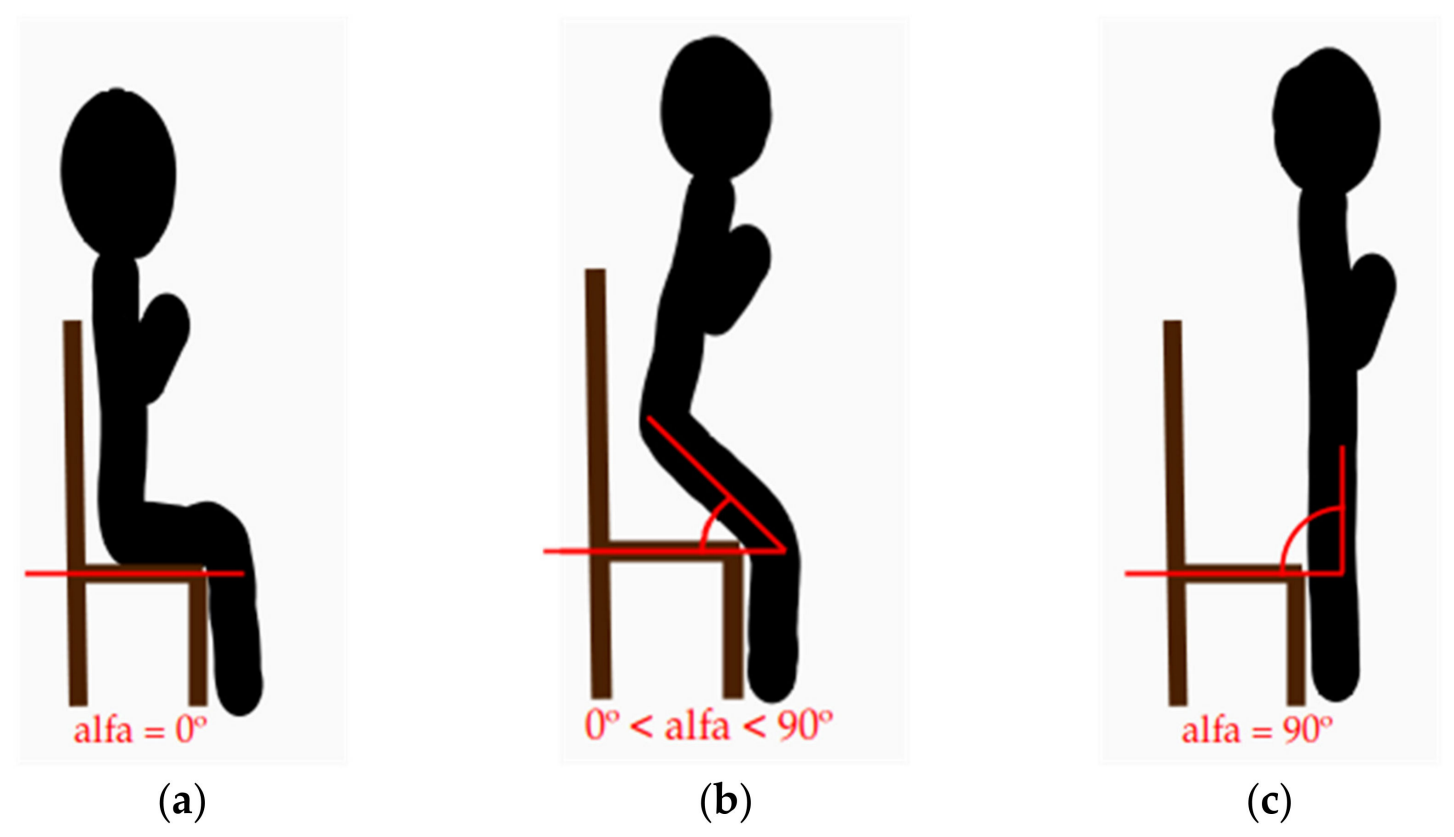
Definition of the thigh angle for the 30-s CST. It is defined as the angle between the subject’s thigh and a horizontal plane perpendicular to gravity (e.g., the seat of the chair). It is depicted as the angle (alfa) between the red line along the longitudinal direction of the subject’s thigh and the red line on the seat of the chair; therefore, (**a**) the expected value of the thigh angle in the sitting position is 0; (**b**) the value of the thigh angle at any time during SiSt and StSi transitions is bigger than 0 and lower than 90°; and (**c**) the expected value of the thigh angle in the upright position is 90°.

The STS analysis algorithm itself involves two steps. First, the thigh angle is estimated in real-time as the acceleration samples arrive. The thigh angle in the 30-s CST was defined as the angle between the subject’s thigh and a horizontal plane perpendicular to gravity (e.g., the seat of the chair), as shown in Figure 3b. During a 30-s CST, this angle is expected to vary over time between 0° in the sitting position (Figure 3a) and 90° in the upright position (Figure 3c).

**Figure 4:**
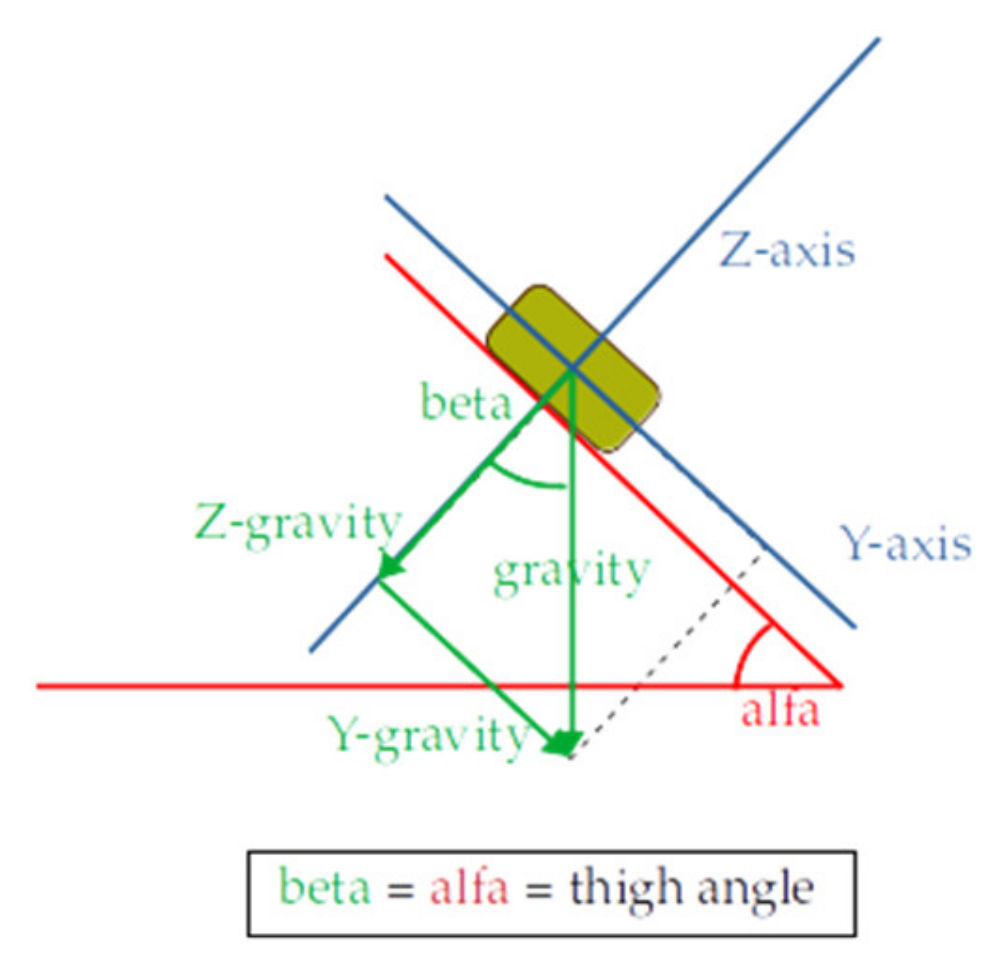
Computation of the thigh angle from the decomposition of gravity into orthogonal components along the axis of the reference system (blue lines) of an accelerometer on the subject’s thigh. According to the convention applied in the preceding figure the thigh angle is represented by the red angle (alfa). Gravity and its components are depicted in green. The green angle (beta) between gravity itself and its Z-component is equal to the thigh angle (alfa), because gravity is always perpendicular to the horizontal plane, and the Z-gravity component is always perpendicular to the thigh.

The thigh angle can then be computed from the gravity readings of the accelerometer on the subject’s thigh, as demonstrated in Figure 4. The thigh angle (red angle, dubbed as alfa) is equal to the angle between gravity itself and the Z-gravity component of the accelerometer readings (green angle, dubbed as beta) because the gravity is always perpendicular to the horizontal plane (the seat of the chair), and the Z-gravity component is always perpendicular to the thigh.

Thus, if the *Y*-axis of the accelerometer is aligned with the thigh itself, as it is in the case of our experimental setting, the angle value at any given moment can be computed from the accelerometer Z-gravity and Y-gravity readings according to the following expression:

(1) $$\alpha =\arctan\left(-g_{y}/g_{z}\right)$$

Obtaining the gravity components from the accelerometer readings would require filtering the raw acceleration signals. However, in order to lower the computational complexity of our algorithm we estimated the thigh angle directly from raw acceleration samples as:

(2) $$\hat{\alpha }=\arctan\left(-a_{y}/a_{z}\right)$$

where a_y_ (i.e., the Y-acceleration component) and a_z_ (i.e., the Z-acceleration component) include the contribution of both gravity and the forces exerted by the subject to execute the SiSt and StSi transitions. The outcome of the expression above is limited by the fact that the tangent function is a periodic function, and the arc tangent function only returns values for the first period of the angle values, i.e., values between −*p*/2 and *p*/2. Theoretically, this should not be a problem because, as stated before, the value of the thigh angle is expected to oscillate within that range (between 0 and *p*/2 radians, i.e., between 0° and 90°). However, when the subject is close to the upright position, there are some non-ideal behaviors that should result in an angle estimation bigger than 90°, but will not if we applied Equation (2). For instance, the value of the Z-acceleration component is expected to always have a negative sign, except at the upright position where it is expected to be zero. Nevertheless, nearby the upright position, the noise from the acceleration and deceleration forces exerted by the older adult could alter the Z-acceleration sign, and turn it into a positive value. In that case, Equation (2) does not return a value bigger than *p*/2 but a negative value between −*p*/2 and zero. In order to make a correct estimation of the angle, the sign of the accelerometer readings must be taken into account according to the following expression:

(3) $$\hat{\alpha }=\left\{\begin{array}{c} -\arctan\left(a_{y}/a_{z}\right){,\mathrm{ifa}}_{z}⩽0\\ \pi -\arctan\left(a_{y}/a_{z}\right){,\mathrm{ifa}}_{z}>0 \end{array}\right\}$$

Please note that the sign of the Y-acceleration component cannot be negative while the sign of the Z-acceleration component is positive, unless the device is upside down, because gravity always points downwards.

**Figure 5:**
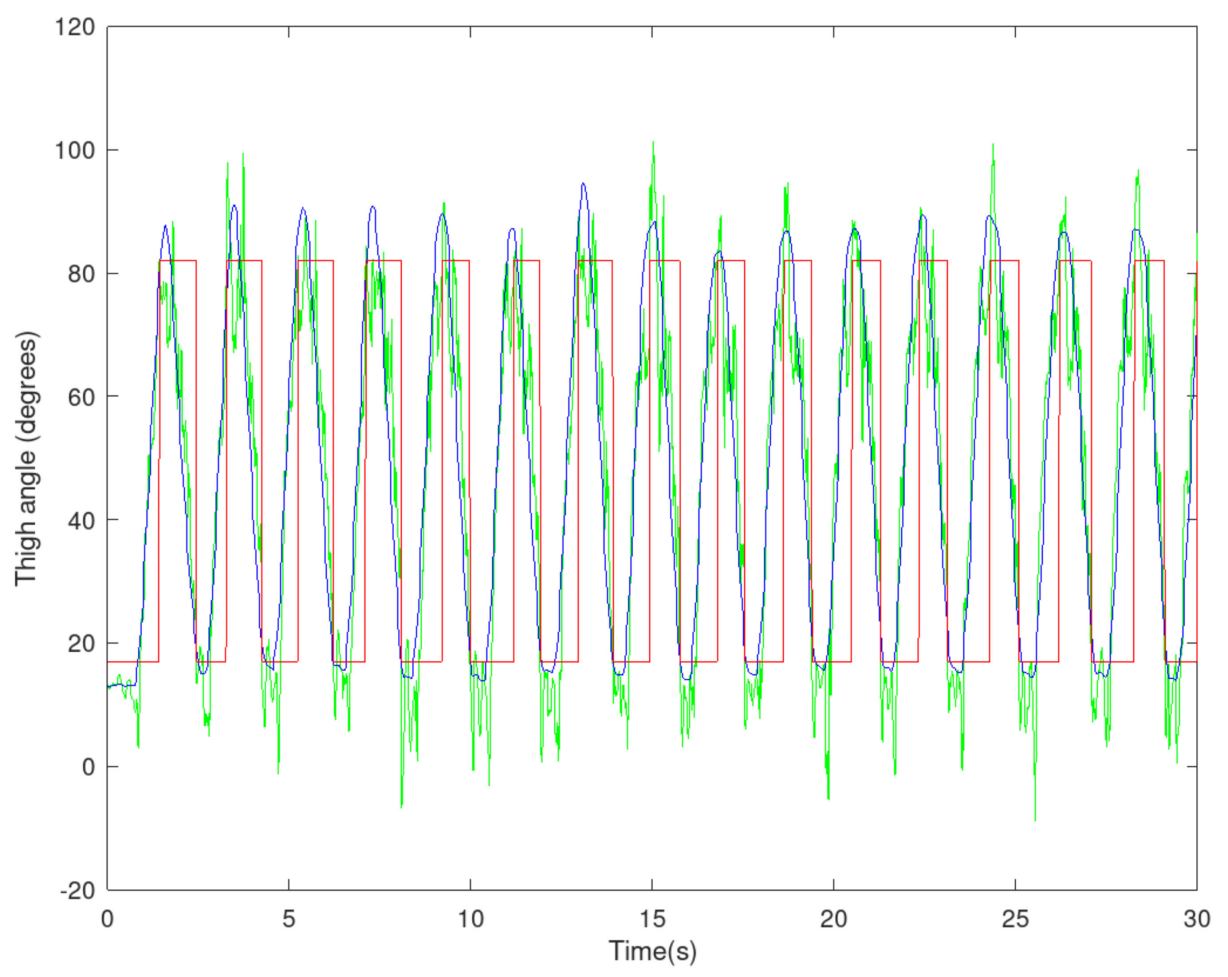
Graphical representation of the outcomes of a 30-s CST. The blue line represents the evolution of the thigh angle over time, computed from the estimation of the gravity components (it is not computed by the device). The green line represents the estimation of the thigh angle used in the device; it is directly computed from raw readings from the accelerometer. The red line represents the output of applying hysteresis thresholding to the green signal.

While the variation of the actual thigh angle over time, and even an estimation based on gravity components, are smooth quasi-periodic signals like the blue line in Figure 5, the values of the thigh angle estimated from raw acceleration readings, and their variation over time, result in a noisy signal like the green line in Figure 5. The said noise is particularly strong close to the maxima and minima of the actual angle, due to the abrupt deceleration forces applied to the sensor upon reaching the upright and sitting positions. Consequently, while the blue signal shows smoothly and clearly defined maxima that can be used to identify the end of a SiSt transition into the upright position, the local maxima and minima in the noisy green signal do not serve that purpose anymore. Which brings us to the second step in the STS analysis algorithm. In the second step, hysteresis thresholding was applied to the signal to remove the effect of the noise in the green signal. The output of such a filter was a binary signal (standing vs. sitting) like the red line in Figure 5. The threshold values and the computational algorithm described below were defined to filter the signal and spot valid SiSt transitions in real time.

The output of the hysteresis thresholding algorithm switches between two different states (i.e., sitting and standing) as follows: The estimated value of the thigh angle is compared to two values configured in a previous stage (see the next paragraph). These two values are known as the sitting-threshold and the standing-threshold. If the previous sample was in a standing state and the current estimated thigh angle reaches a value greater than 0°, and lower than the sitting-threshold, the subject is considered to have completed a StSi transition, the state changes to sitting, and the subsequent SiSt transition is an eligible candidate to count as a valid attempt; otherwise the subsequent SiSt transition will not count as a valid attempt no matter what. On the other hand, if the previous sample was in a sitting state and the extension angle reaches a value greater than the standing-threshold, and lower than 90° during an eligible SiSt transition, the state changes to standing, and the transition counts as a valid attempt; otherwise it is dismissed as a failure.

The rationale behind using the sitting-threshold and the standing-threshold comes from the fact that even though the expected angle values theoretically range from 0° (sitting) to 90° (standing), there are two sources of non-ideal behavior that require the definition of more flexible threshold values. First, mobility constraints may narrow this range for some older subjects. A subject’s readings whose default standing position does not exceed 80°, will never reach the theoretical 90° standing-angle. Thus, valid standing attempts would be dismissed and the automatic count of valid SiSt transitions would result in a wrong score. Analogously, a subject’s readings whose default sitting position does not fall down below 10°, will never reach the theoretical 0° sitting-angle. Thus, subsequent valid standing attempts would be dismissed and the automatic count of valid SiSt transitions would result in a wrong score as well.

The other source of non-ideal behavior is the non-ideal nature of the sensor readings themselves. Even if a subject reaches his default standing position, the sensor might provide a reading slightly lower than the subject’s default standing angle. In such a case a valid attempt would be dismissed as a failure, and the automatic count of SiSt transitions would result in a wrong score. The analogous situation applies to the sitting position and the subject’s default sitting angle.

To avoid the negative impact of these situations on the sensor performance, the sensor is calibrated before initiating a 30-s CST. The subject’s thigh angle in a static sitting position is measured and recorded. In particular, the sitting angle is computed as the mean value of the angle readings collected while the subject is sitting in a static position for four seconds. Then, a correction is applied to allow for some error tolerance. The sitting-threshold is set to its final value by adding 10º to the subject’s default sitting angle. Analogously, the subject’s thigh angle in a static upright position is measured and recorded, and then the standing-threshold is set to its final value by subtracting 10º from the subject’s default standing angle.

##### 2.1.2.3. The Tablet App

The application was developed in Java for Android. The tablet device was a Huawei M2-A01L with Android 5.1.1. The application is used to configure the personalized parameters in the sensor algorithm (i.e., the sitting-threshold and the standing-threshold), to issue a command to the sensor for it to begin the measurement process, and to visualize the test results after completion. The application home screen shows a list of all the sensor devices paired with the tablet so the end-user gets to pick which one to configure. In the case of the data collection stage in the present study, only one device was paired with the tablet. The application has two operation modes, namely, calibration mode and measurement mode. In calibration mode, the values for the sitting-threshold and the standing-threshold are computed and set according to the following process:

1. The researcher puts the sensor into calibration mode by issuing the corresponding command with the app.
2. The researcher asks the sensor to compute sitting angle readings for four seconds and send them back to the app by issuing the corresponding command with the app.
3. The app computes the mean value of these sitting-angle readings and stores them as the subject’s default sitting-angle.
4. The researcher asks the sensor to compute standing-angle readings for four seconds and send them back to the app by issuing the corresponding command with the app.
5. The app computes the mean value of these standing-angle readings and stores them as the subject’s default standing-angle.
6. The researcher enters an error tolerance value for each default angle.
7. The researcher asks the sensor to set the value of the sitting-threshold and the standing-threshold by issuing the corresponding command with the app. The sitting-threshold is computed as the sum of the subject’s default sitting-angle and the error tolerance value for the sitting position. On the other hand, the standing-threshold is computed as the subtraction of the error tolerance value for the standing position from the subject’s default standing-angle.

In measurement mode the application waits for the sensor to send the results of the 30-s CST according to the following process:

1. The researcher puts the sensor into measurement mode by issuing the corresponding command with the app.
2. The researcher asks the sensor to start the 30-s CST measurement sequence by issuing the corresponding command with the app.
3. The application waits idle for the results of the 30-s CST.
4. The application shows the results of the 30-s CST on screen.

#### 2.1.3. Procedure

Seven older subjects were administered a 30-s CST each, in accordance with the following procedure. A member of the research team gave instructions to the subjects to guide them through the process. First, a member of the research team paired the wearable sensor with the tablet device via Bluetooth, and asked the subject to put on the wearable device. The subject was then asked to sit down on the chair to calibrate the sensor sitting-threshold. Next, the subject was asked to stand up to calibrate the sensor standing-threshold. After the calibration stage, the subject was asked to repeatedly stand up from, and sit down on, the chair as fast as possible for 30 s. The subject was asked to do so with his arms folded over his chest, and starting from a sitting position. The sensor emitted a short vibration to indicate to the subject when to start. A trained member of the research team manually counted SiSt transitions. Once the 30 s were over, the sensor emitted another short vibration to signal the subject to stop. Then, the sensor sent the outcomes of the automatic count algorithm to the mobile app, which showed them on screen. A member of the research team took note of the values for the manual and automatic counts.

#### 2.1.4. Analysis

The correlation between the manual and the automatic counts were studied. These two variables are of the interval type, therefore we decided to compute their correlation with Pearson’s moment-product correlation coefficient. Before applying Pearson’s r to the data, the normality of the two data sets (manual vs. automatic counts) was tested. Due to the size of the sample, normality was studied with a Shapiro–Wilk test that resulted not statistically significant in both cases. Therefore, both data sets could be considered to be normally distributed, and we proceeded with Pearson’s r. The Shapiro–Wilk test was calculated using the shapiro.test function, and Pearson’s r was calculated using the cor.test function; in both cases R statistical software, version 3.6.3, was used. The 95% CI for the Pearson’s r estimate was computed by applying Fisher’s transformation.

To further characterize the impact of noise on the sensor performance we studied the statistical significance of our estimation for the probability of finding harmless noise levels in a valid SiSt transition. This situation was modeled as a binomial experiment, where each SiSt transition in the data set corresponds to a binomial event, a correct SiSt identification means harmless noise and, therefore, success, and an incorrect SiSt identification means harmful noise and, therefore, failure. The probability of harmless noise was estimated as the number of correct identifications in the data set divided by the total number of SiSt transitions in the data set. The 95% CI for the probability of success was calculated by applying a binomial test with the binom.test function in the R statistical software, version 3.6.3.

### 2.2. Unsupervised Validation of the Home Care System

#### 2.2.1. Participants

Seven older subjects (3 female and 4 male), between 64 and 74 years old, participated in the unsupervised validation of the home care system. All subjects gave their informed consent for inclusion before they participated in the study. The study was conducted in accordance with the Declaration of Helsinki, and the protocol was approved by the Ethics Committee of the Universidad Politécnica de Madrid on 9 May 2019 (POSITIVE: Maintaining and improving the intrinsic capacity involving primary care and caregivers).

The following inclusion and exclusion criteria were applied:

- A subject COULD ENTER the study if ALL the following INCLUSION CRITERIA applied:
  ◦ The subject is willing and able to give written informed consent for participation in the study.
  ◦ The subject is 64 years old or older.
  ◦ The subject is able to perform the 30-s CST in a safe way.
  ◦ The subject has not been diagnosed with cognitive impairment.
- A subject COULD NOT ENTER the study if ANY of the following EXCLUSION CRITERIA applied:
  ◦ Subjects suffering from any major disability.
  ◦ Subjects suffering from cognitive impairment.

#### 2.2.2. Apparatus

##### 2.2.2.1. The Sensor

**Figure 6:**
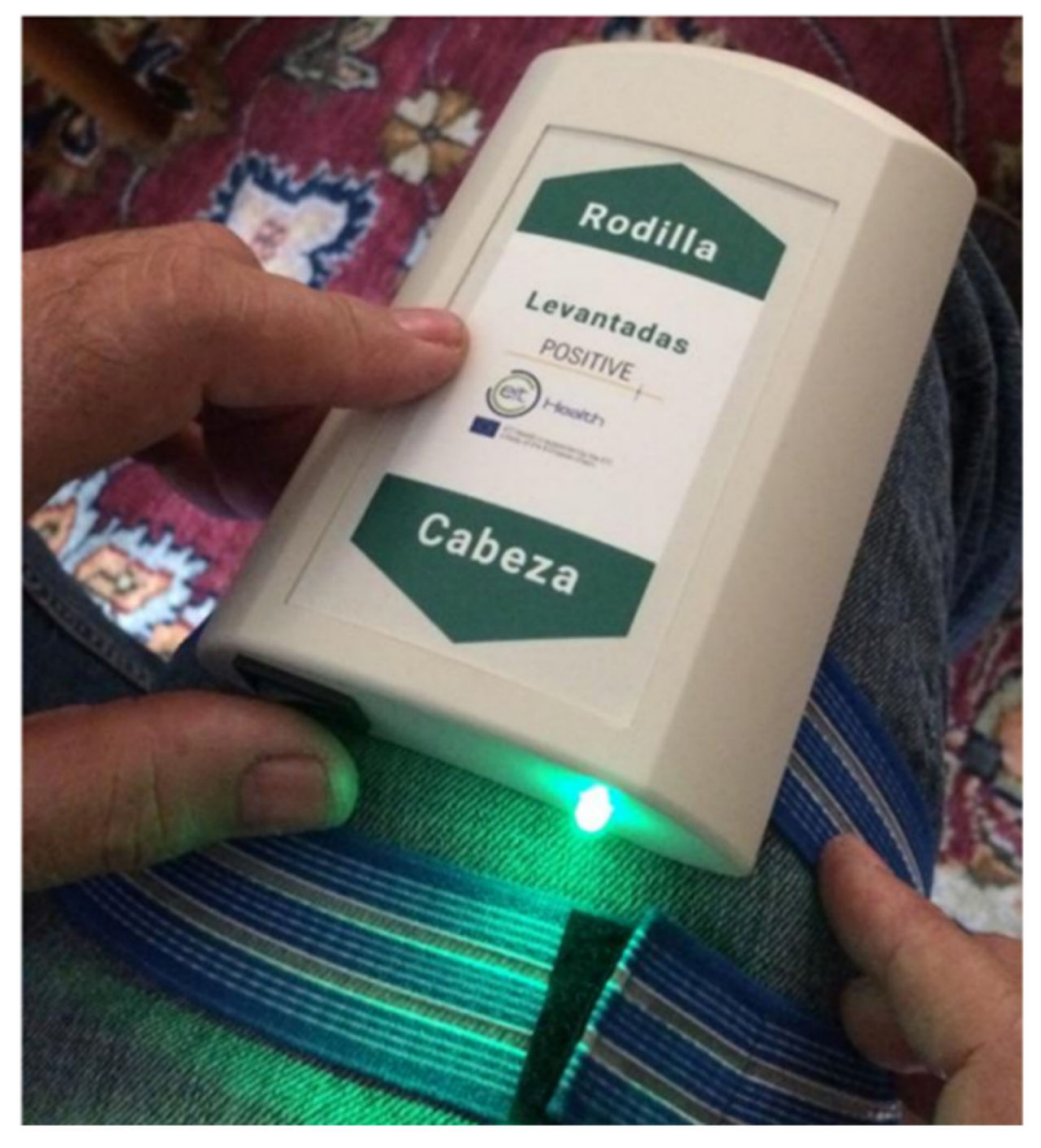
Second version of the sensor casing. The sticker on the sensor reports the correct orientation of the sensor with the help of two tags: “Rodilla”, which is the Spanish word for knee, and “Cabeza”, which is the Spanish word for head.

The sensor hardware was ported to a more ergonomic case which included a sticker with clear instructions about the proper orientation of the ends of the device, with the help of two tags, namely, “Rodilla”, which is the Spanish word for knee, and “Cabeza”, which is the Spanish word for head (see Figure 6).

##### 2.2.2.2. The Home Care Application

The home care application was developed in Java for Android and was a user friendly evolution of the application in Section 2.1.2.3. The application included a user-friendly interface specifically designed for older adults. Once the default and the threshold values of the participants sitting and standing angles were calibrated for the first time, the application recorded the outcomes so it was not necessary to re-calibrate every time the participant took a test. The app provided explanatory pictures, audio, and video to help the participant in preparing for taking a test, and audio instructions were also available to guide him through the whole procedure.

##### 2.2.2.3. The Acceptability and General Impressions Questionnaires

In order to assess the system acceptability, a semi-structured interview comprising the questions in the second column in Table 2 was conducted. Furthermore, the participants’ general impressions were also collected by conducting another semi-structured interview, comprising the questions in the third column in Table 2. The first three questions in each questionnaire are related to the participant’s opinions on the sensor. The remaining ones are related to the participant’s opinions on the application in the tablet.

#### 2.2.3. Procedure

A trained technician went to the participants’ homes to set up the system and explain to them how to use it (only one user per home was configured). The technician delivered a tablet device with the home care application pre-installed. Once in the participant’s dwelling, the technician paired the sensor and the tablet via Bluetooth and proceeded to calibrate the participant’s default sitting and standing angles as described in Section 2.1.2.3. The application recorded the values so the participant did not have to repeat this step every time he took a 30-s CST. The participants could contact the technician to get help to fix any technical issues that could arise over the course of the study. 

The participants used the system for a month. The participant initiates a test by entering into the “My medical tests” in the app. The participants had to follow the instructions of the home care application to complete a 30-s CST, without any supervision or further assistance, and according to the following procedure. The participant put the tablet on a nearby surface. The participant then had to put the device on over his knee, secure it with the strap, and click next on the tablet. Then the participant had to switch on the device and click next on the tablet. After that, the participant had to fold his arms over his chest and wait for the start signal. Then he had to stand up and sit down repeatedly until the stop signal. After that, the participant could switch the device off and take it off. Once the 30-days period of study was over, a member of the research team went to the participants’ homes to pick up the equipment and to conduct an interview to evaluate acceptability and general impressions by administering the corresponding questionnaires.

#### 2.2.4. Analysis

The results of the acceptability and general impressions questionnaires were qualitatively assessed.

## 3. Results

### 3.1. Supervised Validation of the Sensor

Table 3 summarizes the outcomes from the data collection process. Most of the people in older populations are female, and this pattern was also reflected in the composition of the sample of volunteers recruited for the present study (5 female and 2 male).

The mean absolute error for the automatic count of SiSt transitions was computed, as usual, according to the following expression:

(4) $$\mathrm{error}=\frac{1}{7}\cdot \sum_{i=1}^{7}\left|{\mathrm{manualCount}}_{i}-{\mathrm{autoCount}}_{i}\right|.$$

The mean absolute error was equal to zero because the sensor output was error-free in all the seven 30-s CSTs, i.e., all the 67 SiSt transitions were correctly identified. This means the high frequency noise in the estimated angle signal was not larger than the gap between the sitting and the standing thresholds for any of the SiSt transitions in the data set. Therefore, the hysteresis thresholding mechanism had not been affected by said noise and no spurious transitions between states had taken place.

Additionally, the correlation between the manual and the automatic counts was studied to test the statistical significance of the perfect match in our observations. Being two variables of the interval type, we chose Pearson’s r to study their correlation. Before computing Pearson’s r, the normality of the two variables (manual count vs. automatic count) was studied with a Shapiro–Wilk test, as shown in Table 4.

The data set complies with the bivariate normality assumption because the Shapiro–Wilk test resulted not statistically significant for both variables. Therefore, we proceeded to study their correlation with Pearson’s moment–product correlation (r = 1, *p* = 0.00). This correlation estimate showed full correlation between the sensor automatic count and the manual count. The 95% CI was computed as a means to measure the accuracy of our estimation. The cor.test function in R computes the CI by applying Fisher’s transformation, and returned a 95% CI = (1, 1). This result suggests that our observation was indistinguishable from a perfect correlation. However, the Fisher transformation defines the lower and upper limits of the 95% CI as:

(5) $$\tanh\left(\mathrm{artanh}\left(\rho \right)\pm 1.96/\sqrt{\left(n-3\right)}\right),$$

where r is the Pearson correlation coefficient and n is the sample size, which in this case equals the number of participants recruited for the study. The hyperbolic arc tangent function is defined only within the open interval (−1, 1), but not for the case when r equals one. Since the hyperbolic arc tangent function tends to infinity as r tends to one, the contribution of the sample size to the value of the upper and lower values in Fisher’s expression becomes irrelevant. Therefore, we think we are not getting much information about the impact of our sample size on the accuracy of our estimation.

In order to tackle this issue and study the accuracy of our sensor, we studied the probability of finding a SiSt transition with harmless levels of noise; because the higher the number of transitions with harmless levels of noise, the more accurate the sensor outcomes will be. The situation was modeled as a binomial experiment (as described in Section 2.1.4), which resulted in an estimated probability of success *p* = 1 with a 95% CI = (0.96, 1). Therefore, the older adults in our sample are expected to produce SiSt transitions with harmless levels of noise at least 96% of the time. Therefore, our sensor would need to observe 25 SiSt transitions in order to make a mistake due to a high level of noise. Since the mean number of SiSt transitions per 30-s CST in our sample is 9.57, the sensor would make a mistake once every 2.61 tests; thus, in order to observe one wrong score, you need to conduct three tests. In other words, according to our estimated 95% CI, in the worst case scenario, our sensor would provide an error free score for at least 67% of the tests conducted, while the remaining 33% would miss the correct score by one SiSt transition. These results show our sensor to be very accurate, however, it could be argued that a sample of seven older adults is too small to be representative of the many interpersonal differences in the general older population and, therefore, the results might have been poorer if the device had been tested on a wider variety of cases.

A larger sample might have shown cases with higher levels of noise; so we analyzed under which conditions angle signals would be noisier, and tested our algorithm behavior in those conditions. The noise in the angle signal is the result of the acceleration and deceleration forces applied to the sensor, especially upon reaching the upright and sitting positions. The faster, and the more sudden, the stand up and sit down moves are, the stronger these forces will be. On the one hand, subjects would have moved faster if they have completed a higher number of SiSt transitions within the 30 s in the test. On the other hand, given a fixed number of transitions, subjects need a larger momentum for those transitions with a wider range. Therefore, the angle signal is expected to be noisier for 30-s CSTs with a higher number of SiSt transitions and a wider range for the thigh angle. Rikli and Jones identified the normative standard values to use the 30-s CST outcomes to compare an older adult’s performance with the average population [41]. According to these standards, a subject’s performance might be considered to be (i) within, (ii) below, or (iii) over the reference range of the average population [41]. However, the reference ranges have different values depending on gender and age [41]. Thus, two people of the same gender with the same test score but belonging to different age groups need not be considered to have the same level of physical decline; and the same applies to two people of different gender but belonging to the same age group. According to these standards 90% of the men in the younger age group (between 60 and 64 years old) score below 22 [41]. The analogous scores for the remaining age groups in the case of men are lower than 22; as they are in the case of women of all age groups. On the other hand, Rikli and Jones also identified the critical values that predict physical independence until late in life [42]. An older adult scoring above the critical value is considered to be fit enough to remain independent until late in life; conversely an older adult scoring below the critical value is considered to be at risk of becoming dependent and requires taking action. These critical values depend on gender and age as well [42]. The critical value for men in the younger group (between 60 and 64 years old) is 17 [42]. The critical values for the remaining age groups in the case of men are lower than 17; as they are in the case of women for all age groups. Thus, we took 22 SiSt transitions as a reference value for an extreme and highly demanding scenario, and 17 SiSt transitions for a critical and likely scenario. Then, we conducted an exploratory study to inquire about the performance of our approach under those two scenarios.

**Figure 7:**
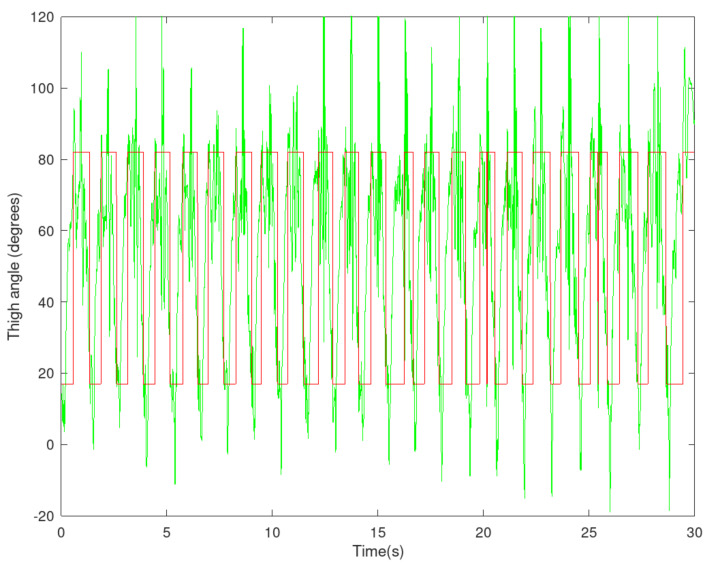
Graphical representation of the outcomes of a 30-s CST in the presence of some transitions with harmful levels of noise. The green line represents the estimation of the thigh angle over time, while the red line represents the transitions detected by the hysteresis algorithm. The transitions around Time = 20 and Time = 25 show a strong and narrow inverse peak of noise that tricks the algorithm into detecting a spurious StSi transition, and then another spurious SiSt transition. Thus, an extra SiSt transition was detected for each of them, and the final score was overestimated by two points; 25 SiSt transitions were reported instead of 23.

A member of the research team took ten 30-s CSTs scoring 22 or above (highly demanding scenario) and another ten 30-s CSTs scoring around 17 (critical scenario). The data for this exploratory study were collected with a smartphone (Nokia 6 TA-1021 with Android 9) on the subject’s thigh and were processed with GNU Octave 5.2.0; this was because the researchers were locked down at their homes, due to the COVID-19 pandemic, and did not have access to the prototypes of the sensor devices. The experiment in the highly demanding scenario resulted in a total of 230 SiSt transitions. Of which, 15 showed harmful noise. All 15 behaved like the transitions depicted around Time = 20 and Time = 25 s in Figure 7. Both transitions show a strong and narrow inverse peak of noise (green line in Figure 7) that tricks the algorithm into detecting a spurious StSi transition, and another spurious SiSt transition (red line in Figure 7). Thus, an extra SiSt transition was detected for each valid transition affected by this kind of noise; in the case of Figure 7, the final score was overestimated by two points, i.e., 25 SiSt transitions were reported instead of 23. All the signals collected, and the code to process and visualize them, are available as Supplementary Materials.

Like in the case of the older adults’ data set, we studied the probability of finding a SiSt transition with harmless levels of noise. Again, it was modeled as a binomial experiment (as described in Section 2.1.4) and the experiment resulted in an estimated probability of success *p* = 0.93, with a 95% CI = (0.89, 0.96). According to the lower limit of this CI, it would be necessary to observe 10 SiSt transitions in order to observe one of them with a high level of noise. The mean number of SiSt transitions per 30-s CST in our sample is 23; thus, between two and three high level noise transitions would be observed per test. On the other hand, according to the upper limit of the 95% CI, i.e., *p* (harmless noise) = 0.96, only one in two tests would miss the correct score, and would do so by a single point.

Finally, the experiment in the critical scenario resulted in a total of 173 SiSt transitions. The algorithm successfully identified all of them. In this case, the estimated probability of success is *p* = 1 with a 95% CI = (0.98, 1). Therefore in the worst case (lower limit of the CI), it is necessary to observe 50 SiSt transitions in order to observe one error. Since the mean number of SiSt transitions in our sample is 17.3, an error would be observed every 2.89 tests. Thus, in order to observe one wrong score, you need to conduct three tests. Therefore, according to the 95% CI, the noise pattern around the critical value would result in a single wrong transition in only one in three tests. Under such a noise pattern our sensor would remain very accurate around the target critical value.

### 3.2. Unsupervised Validation of the Home Care System

#### 3.2.1. The Acceptability Questionnaire

##### 3.2.1.1. Question #1: What Difficulties Did You Find While Using the Sensor?

All but one of the participants declared they did not find any major problems while using the device. One participant declared having experienced some pain in his knee due to osteoarthritis. The same participant also declared being worried about the possibility of the device falling out during the course of the 30-s CST.

##### 3.2.1.2. Question #2: What is Your Opinion on the Sensor?

All the participants provided favorable answers to this question. They highlighted that the sensor is comfortable and easy to use. They also remarked that the labels on the sensor sticker were easy to follow and helped them to know how to correctly put the device on the leg.

##### 3.2.1.3. Question #3: How Did You Feel While Using the Sensor?

The participants declared that they felt comfortable using the device, and that they felt motivated to improve their performance over time. In line with the answers to Question #1, one participant declared to be worried about the possibility of the device falling out during the course of the tests.

##### 3.2.1.4. Question #4: What Difficulties Did You Find While Using the Tablet?

Some participants struggled to understand the video instructions, and some participants faced a few technical issues, however, they declared not to have experienced any major problems once those issues were fixed.

##### 3.2.1.5. Question #5: What is Your Opinion on the Tablet?

Most users reported satisfactory experiences and good opinions on the tablet.

##### 3.2.1.6. Question #6: How Did You Feel While Using the Tablet?

Some participants experienced some technical issues that made them feel uncomfortable. However, their experience and feelings became more satisfactory once these issues were solved.

#### 3.2.2. The General Impressions Questionnaire

##### 3.2.2.1. Question #1: Was the Device Easy to Put on?

Six participants reported they found the device easy to put on. None of the participants reported that they did not find it easy, however, one of them reported the strap did not fit very well around his leg.

##### 3.2.2.2. Question #2: Do You Find the Device Comfortable?

Six participants reported they found the device comfortable. In line with the answers to Question #1, one of them reported some issues regarding the length of the strap.

##### 3.2.2.3. Question #3: Do You Think You Would Be Able to Use the Device at Home on Your Own?

All the participants felt able to use the device at home on their own.

##### 3.2.2.4. Question #4: What Activities Did You Find the Most Difficult to Achieve While Using the Tablet?

Some participants detected some discrepancies between the explanatory videos and the textual description of the exercise, which made them have some doubts. The rest of the application features were reported to be easy to understand.

##### 3.2.2.5. Question #5: Which Features Did You Find the Hardest to Understand in the Tablet?

In line with the answers to Question #4, all of the application functionality was reported to be easy to understand.

##### 3.2.2.6. Question #6: What are Your General Impressions on the Tablet?

All the participants reported favorable opinions about the tablet. They highlighted the motivating potential of such an app and the subsequent health benefits.

##### 3.2.2.7. Question #7: Do You Think You Would Be Able to Use the App at Home on Your Own?

All the participants felt able to use the app at home on their own. However, one of them highlighted that he would be able to do so as long as the application did not become more complex, and another one highlighted that technical support would be required.

## 4. Discussion

Our sensor took advantage of the quasiperiodic variation of the thigh angle over time (i.e., the angle between the longitudinal axis of the subject’s thigh and a horizontal plane perpendicular to gravity, e.g., the seat of the chair). The thigh angle was computed from acceleration readings from an accelerometer on the subject’s thigh. Previous works found in the literature have taken advantage of the quasiperiodic variation of some other variables such as the trunk pitch-angle [23], vertical velocity [23,24], and vertical position [24] to study repetitive STS cycles in STS5 and 30-s CST tests. They estimated the values of these variables from the readings of an IMU on the L3 region of the subject’s lumbar spine. We think the thigh angle is a more convenient approach for two reasons. On the one hand, we think that older people might find it easier to correctly place the sensor on their thighs than on their lower backs, especially if they do not have any help to put them on. However, none of the papers studied their algorithm’s sensitivity to misplacing of the sensor. The procedure for the estimation of the thigh angle described in the present paper (i.e., estimating the angle from the Y-acceleration and Z-acceleration components) requires the sensor *X*-axis to be aligned with the knee rotation axis. However, this constraint is easy to overcome by extending the angle estimation expression to its three-dimensional form. On the other hand, computing trunk pitch-angle, vertical velocity, and vertical position require integration and even double integration of the IMU readings. Due to the noisy nature of the latter, the result is distorted by drift and requires a lot of effort to estimate the original signal with computationally complex algorithms. Millor et al., for instance, applied double integration, combined with fourth-level polynomial curve adjustment and cubic splines interpolation [24]. Conversely, it is not necessary to integrate the acceleration readings to make an estimation of the thigh angle. Such an estimation can be computed from the values of the different components of gravity in the accelerometer reference system; and these values can be estimated by filtering raw acceleration readings.

We did not include any Kalman or complementary filters in our design to avoid the extra hardware and computational load. Instead, we estimated the thigh angle directly from raw acceleration readings; which resulted in noisy but drift-free angle estimations. In spite of the noisy nature of the angle estimations, the device showed an excellent performance. All the SiSt transitions were correctly identified in real time, and the device provided error-free outcomes for all the seven 30-s CST conducted with older adults. The narrow CI returned by the cor.test function in R suggests this observation is indeed statistically indistinguishable from a perfect correlation. However, the limitations described for the Fisher transformation in Section 3, to accommodate such an extreme value for Pearson’s r, make us think we are not getting much information about the impact of our sample size on the accuracy of our estimated correlation.

Looking at the results of our study from a different perspective, we studied the accuracy of our device based on the probability of observing noise levels high enough to exceed the gap between their personalized upper and lower thresholds in the hysteresis stage. Since all the transitions were correctly identified in real time, we concluded that the participants in the study did not generate any transitions with such high levels of noise. According to the narrow 95% CI in our estimation, low levels of noise are expected to happen at least 96% of the time. Which would result in very accurate sensor outcomes. This result can be generalized to the SiSt transitions generated by any population represented by our sample. However, our sample is limited because it could be argued that seven older adults are too few to be representative of the many interpersonal differences in the general older population and, therefore, signals with a higher level of noise might have been observed if the device had been tested on a wider variety of cases. Nevertheless, we did not find any SiSt transitions with harmful levels of noise in our exploratory study for the critical scenario (around 17 transitions with high momentum). According to the narrow 95% CI obtained for that critical scenario (0.98, 1), even if any high noise transitions were to be observed, a single wrong transition would be observed in only one in three tests. Since the 30-s CST is expected to be scheduled to be taken once or twice a week in a home care scenario, we did not observe any risks of missing anyone not fit enough due to sustained overestimated scores over time. We observed that a frequent overestimation of the scores is likely to happen in the highly demanding scenario (over 22 transitions with high speed and high momentum). However, we think that this result does not have a strong impact on the utility of our approach for two reasons. First, less than 10% of the older population are able to reach such high scores, and second, even in case of overestimation, people scoring over 22 are far away from the critical threshold, and therefore are undoubtedly fit enough not to require any immediate intervention. Anyway, further experiments may be useful to characterize the noise profile between the scores of 17 and 22.

We integrated the sensor into a home care app that guides the user throughout the process of taking a 30-s CST, and conducted an acceptability study with older adults in free-living conditions (i.e., using a home care app at home for several weeks to interact with the device without any assistance). All the participants kept using the system throughout the course of the study and none of them dropped out. This observation is in line with their favorable opinions about both the sensor and the application; and, in particular, corroborates the participants’ positive answers to whether they feel able to use the system at home on their own. Despite the excellent results of this acceptability study, further studies will be necessary to test, on the one hand, the long-term acceptability and adoption by older adults and their caregivers, and, on the other hand, to test the feasibility of this novel home care model for frailty in accommodating end-users’ needs and expectations, not just on the older adults’ side, but also on the health care professionals’ side.

## 5. Conclusions

We developed a system for older people to self-administer the 30-s chair stand test (CST) at home without supervision. The system comprises a low-cost sensor that automatically detects and counts sit-to-stand (SiSt) transitions in real time, and a home care application that guides older people through the whole procedure. We studied whether such a system was able to match older people’s abilities and expectations so they can use it at home on their own without any supervision. The sensor automatic counts were perfectly correlated to the researcher’s manual count, so we concluded that the signals generated by the participants did not push the device to its operational limits. This observation is supported by a very narrow 95% CI for the probability of finding a SiSt transition with a low level of noise. The small size of our sample limits our ability to generalize this result to the general older population because more demanding signals might have been observed if the device had been tested on a larger sample. However, we did not find harmful levels of noise in any of the signals in our exploratory study around the critical score. Thus, we did not observe any risks of missing anyone not fit enough due to sustained overestimated scores over time. None of the participants in the unsupervised study of the complete system dropped out, and at the end of the study none of them reported any major problems in understanding the system and interacting with it. They declared they felt comfortable using it, and felt able to use it on their own. Thus, the system is suitable to be used by older adults in their homes without professional supervision.

## Figures and Tables

**Table 1:** The phenotypic model for frailty involves the assessment of five different components (weight loss, exhaustion, weakness, slowness, and low physical activity); and then diagnosing the subject as robust, pre-frail, or frail, according to the number of components that resulted positive in the tests: robust (green rows) for zero components, pre-frail (yellow rows) for 1 or 2 components, or frail (red rows) for 3, 4, or 5 components.

If a Subject Tests Positive to:	He is Diagnosed as:
0 components	Robust.
1 component	Pre-frail.
2 components	
3 components	Frail.
4 components	
5 components	

**Table 2:** List of questions in the acceptability and general impressions questionnaires. The first three questions in each questionnaire ask about the participant’s experience while using the sensor. Whereas, the remaining ones ask about the participant’s experience while using the home care application on the tablet.

Type of Question	Acceptability Questionnaire	General Impressions Questionnaire
Related to the sensor	1. What difficulties did you find while using the sensor?	1. Was the device easy to put on?
	2. What is your opinion on the sensor?	2. Do you find the device comfortable?
	3. How did you feel while using the sensor?	3. Do you think you would be able to use the device at home on your own?
Related to the application	4. What difficulties did you find while using the tablet?	4. Which activities did you find the most difficult to achieve while using the tablet?
	5. What is your opinion on the tablet?	5. Which features did you find the hardest to understand in the tablet?
	6. How did you feel while using the tablet?	6. What are your general impressions on the tablet?
		7. Do you think you would be able to use the app at home on your own?

**Table 3:** Data collected during the experimental procedure. Each row in the table holds the data for one of the seven older adults in the experiment (age: m = 83.29 years old, sd = 4.19; gender: 5 female and 2 male). Each participant took a single 30-s CST. The outcomes from the sensor automatic count match the outcomes from the trained professional’s manual count for all the seven participants. All the 67 SiSt transitions that took place were correctly identified, and the mean absolute error was equal to zero.

Participant ID	Gender	Age	Default Sitting-Angle	Default Standing-Angle	30 s CST Result (Manual Count)	30 s CST Result (Automatic Count)
1	male	88	25°	55°	5	5
2	female	87	25°	70°	6	6
3	male	81	27°	87°	13	13
4	female	79	25.5°	84°	8	8
5	female	79	18.7°	85°	8	8
6	female	81	44°	81°	9	9
7	female	88	19°	82°	18	18

**Table 4:** Results of the normality test for the manual count data set (left) and the automatic count data set (right). None of the tests were statistically significant. Thus, we did not find statistical significance to state that any of the data sets were not normally distributed. Therefore, we considered that they complied with the bivariate normality assumption, and proceeded to study their correlation with Pearson’s r.

Manual Count	Automatic Count
W = 0.88227, *p*-value = 0.2367	W = 0.88227, *p*-value = 0.2367

## Supplementary Materials

The data collected for the exploratory study on the demanding and the critical scenarios, together with the code used to process and plot the corresponding signals, are available online at https://www.mdpi.com/1424-8220/20/20/5813/s1.
